# The cost and cost-effectiveness of gender-responsive interventions for HIV: a systematic review

**DOI:** 10.7448/IAS.17.1.19228

**Published:** 2014-11-04

**Authors:** Michelle Remme, Mariana Siapka, Anna Vassall, Lori Heise, Jantine Jacobi, Claudia Ahumada, Jill Gay, Charlotte Watts

**Affiliations:** 1 Social and Mathematical Epidemiology (SaME) Group, London School of Hygiene and Tropical Medicine, London, UK; 2 Joint United Nations Programme on HIV/AIDS (UNAIDS), Geneva, Switzerland; 3 Health Policy Project, Futures Group, Washington, DC, USA

**Keywords:** gender equality, gender-based violence, HIV/AIDS, cost, cost-effectiveness, critical enablers, development synergies, investment approaches

## Abstract

**Introduction:**

Harmful gender norms and inequalities, including gender-based violence, are important structural barriers to effective HIV programming. We assess current evidence on what forms of gender-responsive intervention may enhance the effectiveness of basic HIV programmes and be cost-effective.

**Methods:**

Effective intervention models were identified from an existing evidence review (“what works for women”). Based on this, we conducted a systematic review of published and grey literature on the costs and cost-effectiveness of each intervention identified. Where possible, we compared incremental costs and effects.

**Results:**

Our effectiveness search identified 36 publications, reporting on the effectiveness of 22 HIV interventions with a gender focus. Of these, 11 types of interventions had a corresponding/comparable costing or cost-effectiveness study. The findings suggest that couple counselling for the prevention of vertical transmission; gender empowerment, community mobilization, and female condom promotion for female sex workers; expanded female condom distribution for the general population; and post-exposure HIV prophylaxis for rape survivors are cost-effective HIV interventions. Cash transfers for schoolgirls and school support for orphan girls may also be cost-effective in generalized epidemic settings.

**Conclusions:**

There has been limited research to assess the cost-effectiveness of interventions that seek to address women's needs and transform harmful gender norms. Our review identified several promising, cost-effective interventions that merit consideration as critical enablers in HIV investment approaches, as well as highlight that broader gender and development interventions can have positive HIV impacts. By no means an exhaustive package, these represent a first set of interventions to be included in the investment framework.

## Introduction

Three decades into the epidemic, HIV incidence remains persistently high in some regions, and HIV/AIDS is still a leading global cause of morbidity and mortality [1]. Gender inequality is an important driver of the epidemic, particularly in Sub-Saharan Africa, where women and girls represent 58% of people living with HIV [2–4] . Rigid gender roles, along with gender disparities in education and employment, severely limit women's ability to negotiate sex and condom use [5]. In addition, power inequalities between women and men, and the fear or experience of violence may increase HIV vulnerability and limit women's access to HIV services or adherence to HIV prevention or treatment technologies [6–11] . This makes HIV prevention especially difficult for women and girls.

In the context of limited resources and the political commitment to the HIV response, UNAIDS and partners have proposed an investment approach to ensure that resources are invested in the most cost-effective interventions, including for populations most at risk [12]. This “investment framework” advocates for the prioritization of six “basic programmes” that directly reduce HIV transmission, morbidity and mortality. The framework also identifies the importance of complementary “critical enablers” – activities that are necessary to support the effectiveness and efficiency of basic programmes, and need to be funded as part of the HIV response. The potential value of synergistic investments in other health and development sectors that may have HIV-related impacts (“development synergies”) – and could partly be supported through the HIV response – is also stressed [13].

The investment framework has played an important role in framing budget estimates and national-level planning and grant negotiations [14]. Estimates of the global cost of implementing the investment framework were produced in 2011, with the costs of critical enablers being produced by estimating the costs of community mobilization, and then adding a 10% mark-up on the costs of basic programmes, as a rough proxy for the potential costs of other enabling activities [12]. Overall, it has been estimated that critical enablers and development synergies could be allocated over 40% of the HIV resource base, but this is far from the reality at country level [15,16].

Although recognized as important, there has been a lack of clarity regarding how or what gender-responsive interventions should be included in HIV investment approaches [17]. Gender equality and gender-based violence (GBV) programmes were initially classified as development synergies, but are increasingly recognized as also integral to an effective response [13,18]. Given the particular challenges that women and girls face in accessing and benefiting from basic HIV programmes, it is important to assess whether some programme components function as critical enablers and should be more explicitly included in national HIV responses and in the investment framework [19,20].

All programmatic interventions can be classified along a “gender continuum” according to the level of change they seek to achieve [21,22]. We consider an intervention to be “gender-responsive” if it takes into account and addresses the different needs of women/girls and men/boys in its design, or explicitly aims to redress existing inequalities between the sexes. A number of previous reviews have looked at the effectiveness of gender components in selected HIV programme areas, but none have considered their cost-effectiveness [8–10,23–29] . To address this gap, this paper systematically reviews evidence on the costs and cost-effectiveness of effective gender-responsive HIV interventions. In addition, where this has not been done, it seeks to explore the incremental cost and effects of gender-responsive programme components.

## Methods

Our approach consisted of three main steps. We first synthesized existing evidence on effective gender-responsive HIV interventions from low- and middle-income countries, drawing on the findings from an existing review on what HIV interventions work for women [30]. For the effective interventions identified, we then conducted a systematic review of their costs and cost-effectiveness. Finally, where feasible, comparisons were made between costs and effects to obtain measures of the potential incremental impacts attributable to the inclusion of gender-responsive investments.

### Effectiveness synthesis

Existing evidence on the effectiveness of gender-responsive HIV interventions was extracted from an extensive review conducted with support from the US President's Emergency Plan for AIDS Relief's Gender Technical Working Group and the Open Society Foundations [30]. We used this database (www.whatworksforwomen.org) to identify effective interventions. For each intervention identified, we reviewed the primary source article, and retained studies that met the following inclusion criteria:

1. Effectiveness evidence rated as I to III on an adjusted Grey scale (i.e. Grey scale I for a systematic review, Grey scale II for a randomized controlled trial, Grey scale IIIa for a study without randomization but including a non-equivalent control group, and Grey scale IIIb for a study without randomization or a control group) [30,31].
2. Contained data from a low- or middle-income country.
3. Presented quantitative intervention impact data on either reported HIV-related behaviours or biologically confirmed outcomes (including studies that used reported behavioural data to model impacts on HIV transmission).

When compiling the evidence on effectiveness, we chose to group the interventions identified into three types. The first are gender-responsive activities that can be added to basic HIV programmes to enhance their effectiveness and efficiency by addressing gender-related barriers to behaviour change, service uptake and retention (HIV+). The second comprises HIV-specific interventions that could be added onto gender-responsive development programmes, to achieve a synergistic HIV effect (Gender+). The last type (Gender) consists of gender-responsive development interventions that do not explicitly include programmatic HIV components, but may nevertheless have secondary HIV benefits because of their impact on gender inequalities and/or violence.

### Cost and cost-effectiveness search

To identify all published costing and economic evaluation studies of gender-responsive HIV interventions, we searched PubMed, EconLit, Eldis and HIV, and gender websites, following PRISMA guidance [32]. The terms searched thematically covered 1) HIV/AIDS, 2) gender, 3) intervention, and 4) economic/impact evaluation (see Supplementary appendix for more details).

Articles identified were included if they had been published in English, French or Spanish between 1 January 1990 and 30 June 2014; presented cost or economic evaluation data; and assessed gender-responsive interventions. Additional bibliography searches from review articles were conducted and recommendations from the UNAIDS expert reference group considered (An expert reference group was established by UNAIDS to provide technical guidance to the study, including representatives from other UN organizations, donor agencies, civil society and academia.). Additional articles analyzing intervention effectiveness that were identified through this search were included under the effectiveness category if they met the inclusion criteria mentioned above.

The only exception to the review was data on the integration of sexual and reproductive health and HIV services. Integration is a gender-responsive intervention, as it restructures services to better meet women's needs. However, as a recent systematic review has extracted this cost-effectiveness data [33], we used its findings, but did not repeat the review as part of this project.

After the first title-based screening, citations were downloaded into reference management software (Endnote X3) for a second round of title/abstract screening, conducted by another researcher. Full texts were then read to ensure correct inclusion. Any uncertainty was resolved through discussion among at least two authors.

### Data extraction

A data extraction spreadsheet was developed, capturing intervention and study characteristics. Outcomes related to HIV/STI (sexually transmitted infection) incidence or prevalence, disability or quality-adjusted life years (DALYs/QALYs), proxies of unprotected sex (marriage rates, pregnancies), sexual behaviour, HIV service uptake and risk factors (e.g. experiencing violence) were extracted. For the costing studies, we also extracted detailed information on methods and cost estimates. Costs were adjusted to 2011 United States' Dollars (US$).

### Quality assessment and data synthesis

The quality of the costing and economic evaluation studies was assessed using an adapted version of the *British Medical Journal's* checklist for economic evaluations [34] (see Supplementary appendix for more details). Two reviewers scored study quality independently and discrepancies were resolved through discussion.

Given the diversity of the interventions, outcomes and costing methodologies, we adopted a narrative approach to data synthesis [35].

### Analyzing the return on investment

Interventions with a cost per DALY averted or QALY gained below the country's per capita gross domestic product (GDP) [36] were considered cost-effective, as per the lower threshold of WHO [37]. Where a cost per HIV infection averted was estimated, we translated this into a cost per DALY averted for antiretroviral therapy (ART) and no-ART scenarios, using standard DALY formulae and country-specific life expectancy at birth from WHO, as described elsewhere [38,39]. For studies that did not estimate cost-effectiveness ratios (CERs) that could be compared to the WHO threshold, costs were analyzed alongside effects from the same study and/or studies from the same country.

Where the intervention was clearly an incremental investment on a basic HIV programme or an HIV component of a gender-responsive health or development programme, the cost markup was estimated using internally consistent data from the same study, where possible. Otherwise, approximate cost markups were estimated by comparing the unit cost of the gender-responsive component to the country-specific cost used in the investment framework for the specific basic HIV programme or development synergy [12].

## Results

### Evidence of effectiveness

**Figure 1:**
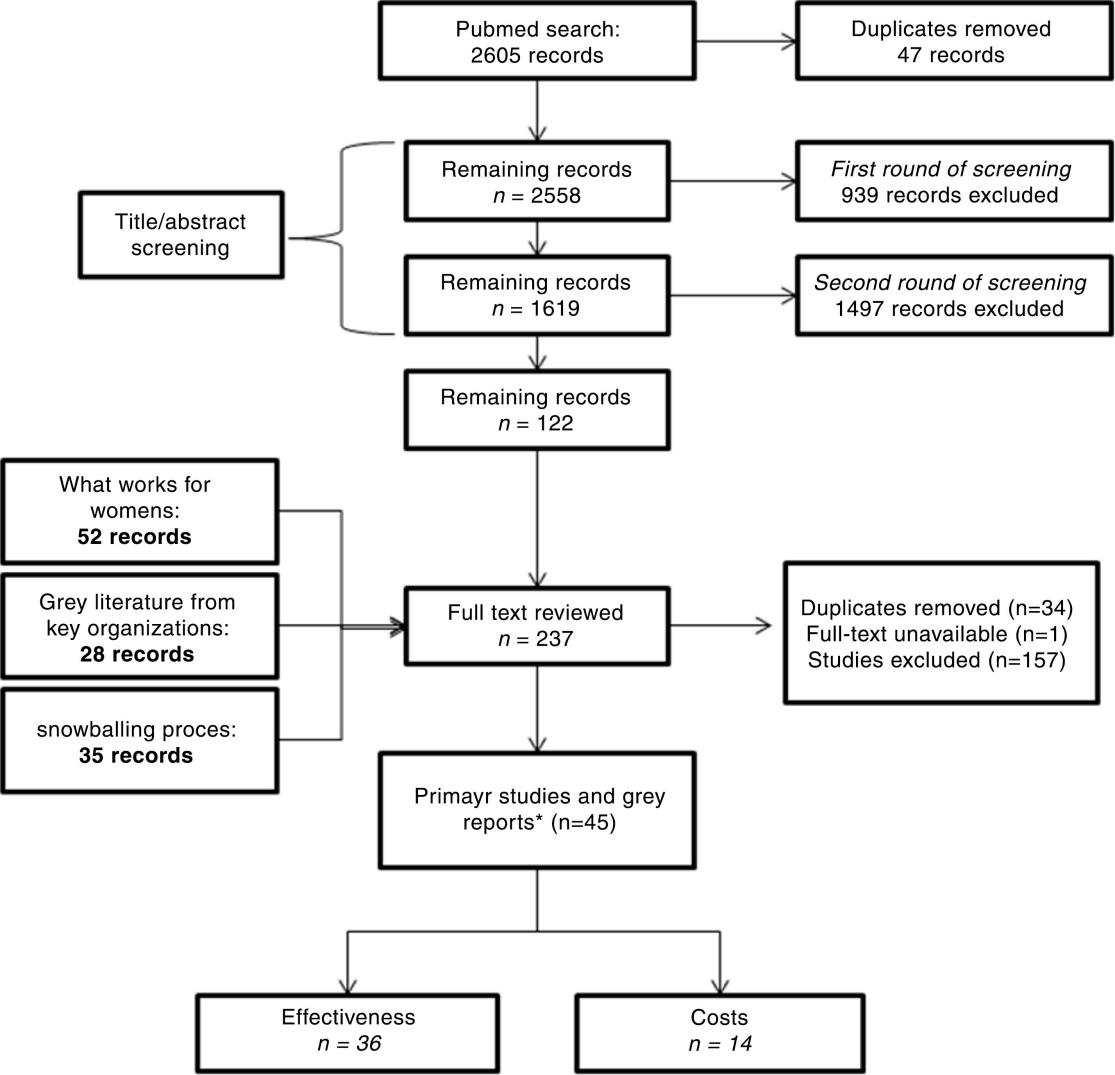
Flowchart for the selection of studies. *The number of studies does not add up because % studies include both effectiveness and cost data.

The effectiveness search identified 36 publications describing 22 gender-responsive interventions found to be effective for HIV (Figure 1). Most of the studies [25] evaluated interventions in countries with generalized epidemics. Interventions for key populations and those focussed on young men came primarily from concentrated epidemics. Thirteen interventions had at least one randomized controlled trial demonstrating HIV-related effects, whereas nine interventions were assessed without randomization, without a control group, or through modelling. In addition, only nine intervention models had been evaluated in multiple trials/studies. Only three studies assessed impact on HIV or other biological outcomes (Herpes simplex virus type 2 (HSV-2) and pregnancy) [40–42] , with the majority considering proximal determinants of HIV risk (namely reported behaviours and experiences).

Gender-responsive activities for programmes to prevent vertical transmission included promoting male involvement through couple counselling [43–46] and training women living with HIV peer support groups [47–49] , which enhanced the uptake and adherence to these prevention services, thereby averting more infant infections [50].

In terms of key population programmes, effective gender-responsive components were identified for female sex workers (FSWs) and people who inject drugs (PWID). The former included community mobilization to prevent violence and promote gender empowerment through collectivization and stakeholder engagement [51–53] ; educational sessions [54,55]; female condom promotion [56,57]; and micro-enterprise services [58,59]. An enhanced gender-responsive service package for FSWs with reported substance abuse, including woman-focussed personalized risk assessments and risk-reduction strategies, increased reported condom use and reduced client and intimate partner violence (IPV) [60,61]. Couple-based educational sessions for PWID were an effective gender-responsive programme component, increasing reported condom use and safe injections [62].

In terms of condom promotion, modelling suggests that expanded female condom promotion and distribution to increase availability could increase consistent condom use among the general population [63]. Another study of a gender-responsive condom negotiation training intervention for married women in Zimbabwe also found improved ability to negotiate increased condom use [64] (Table 1).

**Table 1:** Summary of effectiveness studies identified

	Intervention/programme and study	Location/site and study population	Intervention description and study design (Grey scale^a^)	HIV-related outcome(s)^b^	Size and period of effect	Incremental interpretation
Prevention of vertical transmission	**Promoting male partner participation through individual and/or couple VCT** Becker *et al*., 2009	Tanzania (urban) 3 ANC clinics 1521 women attending ANC, of which 81 were HIV-positive reached at follow-up (51 in the individual VCT arm; 30 in the couples VCT arm)	**HIV+: Invitation of male partner for couples VCT** Pregnant women were given invitation letters for their husbands to come with them for couples VCT at the next ANC visit. Randomized controlled trial in which women in the control arm received individual VCT upon recruitment (Grey scale II)	HIV-positive women receiving Nevirapine for (1) themselves and (2) for their infants	Percentages at follow-up visit three months after delivery date (couples VCT vs. individual VCT): 1) 55% vs. 24% 2) 55% vs. 22% (significant at *p<*0.10)	The study analyzed the incremental effect of couples VCT on the use of protective measures against sexual transmission and uptake of vertical transmission prevention services.
	Farquhar *et al*., 2004	Kenya (urban) 1 clinicAmong 2104 women accepting testing, 308 had partners participate in VCT, of whom 116 were couple counselled	**HIV+: Staff encourage return with partners and couples counselling** Cohort study with a control group, comparing HIV-positive pregnant women whose partners were invited to come to the clinic for VCT (1), those whose partners came for VCT (2) and those who were counselled as a couple (3)(Grey scale IIIa)	Women reporting condom use since last visit Women returning for post-partum follow-up and reporting nevirapine use at delivery Women choosing to breastfeed their infants	Odds ratios at six months post-partum follow-up: 4.2 (1.5–11.5) 3.4 (1.3–9) 0.2 (0.04–0.9)	The study analyzed both the incremental effects of partners coming for individual VCT and for couples VCT on the uptake of vertical transmission prevention services and recommendations.
	Mohlala *et al*., 2011	South Africa 1000 pregnant women (500 in each arm)	**HIV+: Invitation of male partner for ANC and VCT** Invitation letters were provided to women in antenatal care (ANC) for their male sexual partners to attend ANC and VCT. Randomized controlled trial in which women in the control group received invitation letters for their male sexual partners to attend ANC and pregnancy information sessions (Grey scale II)	Male sexual partner that underwent HIV testing Reported unprotected sex in previous 2 weeks	Risk ratios at 12 weeks post-randomization follow-up: 2.82 (2.14–3.72) 0.30 (0.22–0.42)	Not incremental to standard vertical transmission prevention programmes, since the control involved male involvement in pregnancy information sessions. The study analyzed the incremental effect of the VCT invitation letter on ANC attendance, VCT uptake and unprotected sex.
	Msuya *et al*., 2008	Tanzania (urban) district primary healthcare clinics Of 2654 pregnant women asked to invite their partners, 332 male partners came for HIV counselling and testing at the clinics	**HIV+: Invitation of male partner for individual and couples VCT** Pregnant women in third trimester encouraged to inform and invite their partners for VCT. Cohort study comparing pregnant women (HIV-positive and HIV-negative) whose partners came for VCT and those whose did not (control group) (Grey scale IIIa)	HIV-positive women choosing not to breastfeed (recommended at time of study) HIV-positive women adhering to infant feeding method selected at post-test	Odds ratios at two-year follow-up: 3.72 (1.19–11.63) 5.15 (2.18–12.16)	The study analyzed the incremental effect of partners coming for VCT (individual and/or couple) on uptake of vertical transmission prevention services.
	**Peer support groups for pregnant women/mothers living with HIV** Futterman *et al*., 2012	South Africa (peri-urban) 2 NGO and public sites 160 pregnant women attending the clinics who were diagnosed HIV-positive	**HIV+: peer mentoring and cognitive behavioural training (Mamekhaya programme)** Mothers living with HIV (MLH) were linked to mentor mothers who were also HIV-positive and had been trained to provide support.	Centre for Epidemiologic Studies Depression Scale Social support availability scale	Random intercept regression model coefficient (standard error) after six months: 4.43 (1.62) 9.32 (3.53)	The study analyzed the incremental effect of the Mamekhaya programme on uptake of vertical transmission prevention services and adherence to preventive practices.
			Non-randomized trial with control group receiving standard vertical transmission prevention care from medical staff (Grey scale IIIa)			
	Nguyen *et al*., 2009	Vietnam (urban) Referral network among 26 health facilities 30 women (members of the group) who had learned they were HIV-positive before or during a pregnancy and chose to complete the pregnancy	**HIV+: peer support group for HIV-positive mothers** In the self-help group, core members (peer counsellors) delivered publicity materials to create a referral network among health facilities, visited hospitals and VCT sites to make informal contact with potential members. Pre-/post-assessment without control group (Grey scale IIIb)	Women with record for health follow-up at ART sites Women receiving ART (when needed)	Indicators upon joining group and two years after joining the group: Increase from 1/30 to 30/30 Increase from 1/9 to 15/15	The study analyzed the incremental effect of participating in the support group on access to ART.
	Rotheram-Borus *et al*., 2014	South Africa (rural and urban) 4 control clinics with 656 WLH enrolled 4 intervention clinics with 544 WLH enrolled	**HIV+: peer mentoring and support sessions** Pregnant women living with HIV (WLH) were invited to attend 8 meetings with peers, facilitated by peer mentors, and covering various topics, such as normalizing being a WLH, healthy lifestyles, treatment adherence, infant feeding methods and bonding, couple counselling and condom use. Cluster randomized controlled trial with WLH in control clinics receiving standard vertical transmission prevention services (Grey scale II)	Infant fed using one feeding method for first six months Infant weight-for-age *z*-score ≥2 Infant exclusively breastfed for at least six months Mothers not depressed (GHQ<7)	Estimated odds ratio from birth to 12 months post-birth: 3.02 (1.20–7.60) 1.08 (1.01–1.16) 2.38 (1.04–5.44) 1.08 (1.03–1.13)	The study analyzed the incremental effect of receiving peer mentoring and support on service uptake, maternal and child health outcomes.
Key populations – FSWs	**Gender empowerment community mobilization intervention among FSWs** Basu *et al*., 2004	India 2 urban centres 200 brothel-based FSWs (100 in each arm)	**HIV+: integrated empowerment intervention (Sonagachi)** Local peer educators were trained to build skills and confidence in providing education and to foster empowerment and advocacy for local sex workers. The team engaged in ongoing advocacy activities with local stakeholders and power brokers who exerted control over the sex workers’ lives. Randomized controlled trial (Grey scale II)	Proportion reporting 100% condom use Proportion of consistent condom users	Effect after 15 months: 39% increase vs. 11% increase 25% increase vs. 16% decrease	The study analyzed the incremental effect of the Sonagachi model on condom use.
	Markosyan *et al*., 2010	Armenia (urban) 1 public site 120 FSWs	**HIV+: gender empowerment intervention** A health educator implemented a 2-h intervention emphasizing gender-empowerment, self-efficacy to persuade clients to use condoms, condom application skills, and eroticizing safer sex. Randomized controlled trial (Grey scale II)	Consistent condom use (clients in general)	Adjusted odds ratios at six-month follow-up: 2.85 (1.41–5.75)	The study did not consider the incremental effect of the gender-responsive intervention above a standard FSW programme. Instead, it analyzed the effect of the intervention (compared to a do-nothing alterative) on condom use.
	Beattie *et al*., 2010	India (urban) 4 NGOs Over 60,000 FSWs; 3852 participated in IBBA surveys in 4 districts; and 7638 FSWs participated in 691 polling booth surveys in 13 districts	**HIV+: multi-layered violence prevention strategy (Avahan – India AIDS initiative)** A multi-layered district-wide strategy involving policy makers, secondary stakeholders (police officers, human rights lawyers, journalists) and primary stakeholders (FSWs) to stem and address violence against the sex worker community as part of a wider HIV prevention programme. Pre-/post-assessment without control (Grey scale IIIb)	Experience of violence (beaten or raped) in the past year	Adjusted odds ratio after 33 to 37 months: 0.70 (0.53, 0.93)	Not incremental to standard FSW programme. The study analyzed the association between programme exposure (contacted by a peer educator or having visited the project sexual health clinic) and experience of violence.
	**Relationship-based sessions to reduce violence against FSWs** Carlson *et al*., 2012	Mongolia (urban) 166 FSWs engaging in harmful alcohol use	**HIV+: relationship-based sessions, including violence prevention** The interventions consisted of (1) Four-weekly relationship-based HIV/STI risk reduction sessions; (2) Four-weekly enhanced HIV/STI risk reduction intervention with two wrap-around sessions engaging motivational interviewing; (3) Four-weekly sessions on overall health and wellness knowledge and skills. Cluster randomization, with pre-/post-data analysis by intervention group (Grey scale IIIa)	Reported experience of physical or sexual IPV in the past 90 days	Estimated odds ratios at six months follow-up, based on empirical multilevel logistic modelling with an individual-level random effect: 0.46 (0.24–0.88) 0.14 (0.03–0.61) 0.20 (0.096–0.43)	The study does not find an incremental effect of the HIV+ approach on violence, compared to the non-HIV and HIV-specific control interventions.
	**Female condom promotion among FSWs** Thomsen *et al*., 2006	Kenya (urban) 210 FSWs	**HIV+: female condom promotion for FSWs** Adding female condom promotion to a male condom programme providing peer education and IEC materials, as well as distributing female condoms. Pre-/post-evaluation without control group (Grey scale IIIb)	Consistent condom use with all sexual partners in previous 7 days	At 12-month follow-up: Increase from 59.7% before to 67.1% after (*p=*0.04)	The study analyses the incremental effect of female condom promotion on consistent condom use, but given the lack of a control group the effect estimate is not reliable.
	**Micro-enterprise services for FSWs** Odek *et al*., 2009	Kenya (urban) 227 FSWs	**HIV+: micro-enterprise intervention** The micro-enterprise component was added to the existing peer education HIV risk reduction model and consisted of: credit for small business activities, business skills training and mentorship, and promotion of a savings culture. Pre-post design without control group (Grey scale IIIb)	Self-reported weekly mean number of all sexual partners Consistent condom use with regular partners	Mean at 18 to 23 months follow-up: 1.84 (SD 2.15) compared to 3.26 (SD 2.45) at baseline (*p<*0.001) 93.5% compared to 78.9% (*p=*0.031)	The study analyses the incremental effect of micro-enterprise activities in FSW programmes, but given the lack of a control group the effect estimate is not reliable.
	Sherman *et al*., 2010	India (urban) 100 FSWs	**HIV+: micro-enterprise intervention** The micro-enterprise component was added to a standard HIV prevention education intervention, and consisted of 100 h of tailoring training taught by master tailors over the course of a month. The control arm received the same 8 h course on HIV prevention provided twice per week over 2 weeks and provided by 2 health educators. It covered topics around HIV risk, as well as gender, violence and alcohol use. Randomized controlled trial (Grey scale II)	Number of sex partners Number of paying clients per month	Mean at six-month follow-up: 5.0 compared to 11.9 at baseline (*p<*0.001) 3.1 compared to 5.1 at baseline (*p<*0.001)	The study analyses the incremental effect of the micro-enterprise component over and above a gender-responsive HIV prevention education intervention for FSWs.
Key populations – IDUs	**Woman-focussed empowerment-based intervention for high-risk women/FSWs with substance abuse** Wechsberg *et al*., 2006	South Africa (urban) 1 NGO site 93 women who reported recent substance use (cocaine) and sex trading	**HIV+: Woman-focussed sessions, including condom negotiation and violence prevention** The enhanced intervention consisted of 2 one-on-one sessions with a personalized assessment of each woman's drug and sexual risks, information and skills to negotiate condom use, violence prevention strategies and referrals to community resources. Individually randomized controlled trial, with the control group receiving a private 1-h HIV risk reduction education session (Grey scale II)	Male condom used with boyfriend during last sexual encounter Any female condom used with boyfriends in the last month Mean occurrence of victimisation reported by participants Mean number of STI symptoms since last encounter reported by participants	Effect size after one month: RRs at baseline and follow-up were 0.64 and 1.15. Effect size is 0.51 (significant at *p=*0.05) RRs at baseline and follow-up were 0.15 and 1.20, respectively. effect size is 1.15 (significant at *p=*0.01) 4.5 (intervention) vs. 6.3 (control) 0.64 (intervention) vs. 1.07 (control)	Although the intervention targets FSWs, it builds on a basic IDU intervention. The studies analyzed the incremental effect of the woman-focussed intervention on condom use, daily alcohol and drug use, and experience of violence.
	Wechsberg *et al*., 2011	South Africa 583 women		HIV-positive women reporting male or female condom use at last sex Women (HIV-status unknown) reporting male or female condom use at last sex	Odds ratios at six-month follow-up: 7.27 (1.64–32.23) 5.03 (1.26–20.11)	
	**Couple-based prevention for IDUs** Gilbert *et al*., 2010	Kazakhstan 1 site 40 couples that injected drugs in past 90 days (80 participants)	**Health +: couple-based HIV/STI approach** The couple-based HIV/STI risk-reduction intervention (CHSR) included 3 single-gender group sessions with the male and female partners. Randomized controlled trial (Grey scale II)	Proportion of condom-protected acts of vaginal and anal intercourse Proportion of injection acts in which unclean needles or syringes were used in the past 30 days Number of injection acts in which unclean needles or syringes were used in the past 30 days	Regression coefficients (standard errors) at three-month follow-up: 0.19 (0.08) 0.33 (0.05) 12.3 (3.9)	Not incremental effect above standard IDU intervention for HIV, since the control does not cover any HIV topics.
		20 couples per intervention	Random-effects regression analysis			The study analyses the effects of couple-based HIV intervention compared to standard health promotion intervention for IDUs.
Condom promotion and distribution	**Expanded female condom promotion and distribution** Dowdy *et al*., 2006	South Africa Brazil General population	**HIV+: female condom promotion and distribution over and above male condom promotion** Second-generation nitrile female condom (FC2) acquisition, distribution, training and education Impact modelling (Grey scale IIIb)	Fraction of additional sex acts protected by female condoms Incremental HIV infections averted	Assumed to be 3% of the number of male condoms used (low volume), 10% (moderate volume), or 30% (high volume) 1900–32,000 HIV infections averted 100–2000 HIV infections averted	The study models the incremental effect of an expanded country-wide distribution of the second-generation nitrile female condom, over and above existing male and female condom programmes.
	**Condom promotion among married women** Callegari *et al*., 2008	Zimbabwe (urban) 394 sexually active, married women of reproductive age, aged 17 to 47 years	**HIV+: training for married women in condom negotiation and use** A trained counsellor provided a 30-min one-to-one intervention based on social-cognitive models of behaviour change; and a one-month booster session included content similar to enrolment. Pre-/post-evaluation (Grey scale IIIb)	Condom use at last sex Consistent condom use in the past two months	Effect after two months: Increase from 10.1 to 87% Increase from 0.25 to 48.5%	Not incremental to condom distribution, since women in the intervention receive male and female condoms while they may not have had them at baseline. The study analyses the effect of condom negotiation training and condom provision among married women on consistent condom use.
Behaviour Change	**Participatory HIV** **prevention programme** **Building more gender-equitable relationships (stepping stones)** Jewkes *et al*., 2008	South Africa (rural) 70 study clusters comprised 64 villages and 6 townships 1360 men and 1416 women aged 15 to 26 years, who were mostly attending schools	**HIV+: gender-transformative participatory approach with women and men** Intervention stepping stones, a 50 h programme, that aims to improve sexual health by using participatory learning approaches to build knowledge, risk awareness and communication skills, and to stimulate critical reflection. Cluster randomized controlled trial, with the control villages receiving a 3 h intervention on HIV and safer sex (Grey scale II)	Incidence of HSV-2 Men reporting any transactional sex with a casual partner Men reporting problem-drinking	Adjusted odds ratio at 24 months follow-up: 0.67 (0.47–0.97) Adjusted odds ratios at 12 months follow-up: 0.39 (0.17–0.92) 0.68 (0.49–0.94) (not significant at 24 months follow-up)	The study analyses the incremental effect of a more intensive gender-transformative approach on HIV-related risk behaviours.
	**Raising HIV awareness in non-HIV-infected Indian wives (RHANI Wives)** Raj *et al*., 2013	India (urban) 220 women aged 18–40 married to men engaged in heavy drinking or lifetime physical or sexual spousal violence perpetration	**HIV+: educational sessions for married women on sexual communication and empowerment** Multisession intervention focused on safer sex, marital communication, gender inequities and violence. It involved 4 household-based individual sessions and 2 small group-based community sessions delivered over 6 to 9 weeks. Control participants were referred for HIV/STI testing and treatment, local social services for alcoholics and victims of domestic violence. Randomized controlled trial (Grey scale II)	Rate of unprotected sex Condom use at last sex	Risk and odds ratio at 4 to 5 months follow-up: 0.83 (0.75–0.93) 2.42 (1.00–5.70)	The study analyses the incremental effect of this intervention compared to a basic HIV prevention education and referral intervention.
	**Culturally adapted intervention promoting safer sex and relationship control** **(SISTA South Africa)** Wingood *et al*., 2013	South Africa (rural) 5 rural areas 342 isiXhosa women aged 18 to 35 years	**HIV+: educational sessions with women on safe sex and relationship control** SISTA consisted of three 2.5-h interactive group sessions delivered by 2 health educators on consecutive Saturdays at community centres and covering ethnic and gender pride, social and contextual influences that enhance HIV vulnerability and sexual communication skills. The general health comparison condition involved two 2.5-h interactive group sessions covering HIV prevention education, healthy nutrition, hygiene and self-care. Randomized controlled trial (Grey scale II)	Frequency of vaginal sex Frequency of unprotected vaginal intercourse acts in the past 30 days	Adjusted mean difference at six months follow-up: 1.22 (*p=*0.02) 1.06 (*p=*0.02)	The study analyses the incremental effect of a more intensive gender-sensitive approach on HIV-related risk behaviours.
	**Promoting more gender-equitable norms and behaviours among young men** Pulerwitz *et al*., 2006 (Promundo)	Brazil 2 sites+1 control site 508 young men	**HIV+: gender-transformative participatory approach with young men** Two models of the gender-transformative approach were evaluated: (a) interactive group education sessions for young men led by adult male facilitators and (b) group education+community-wide “lifestyle” social marketing campaign to promote condom use using gender-equitable messages. Pre-/post-evaluation with control site (Grey scale IIIa)	Reported STI symptoms over prior three months Condom use at last sex with primary partner	Combination intervention site at six months follow-up: Decreased from 30 to 25% Decreased from 23 to 14% compared to a decrease from 18 to 12% in control (*p<*0.05) Increased from 69 to 70% Increased from 58 to 79% compared to decrease from 64 to 59% in control (*p<*0.05)	Not incremental to basic HIV behaviour change programme.
	Verma *et al*., 2006 (Yaari-Dosti)	India Urban Rural 1423 married and unmarried young men aged 16–29 in urban settings and aged 15–24 in rural settings		Condom use at last sex in the past three months with all partners Reported violence against a partner (either sexual or non-sexual/romantic) in the past three months	Multiple logistic regression odds ratios at six months’ follow-up: 1.913 (*p<*0.001) 2.776 (*p<*0.001) 0.176 (*p<*0.001) 0.502 (*p<*0.001)	Not incremental to basic HIV behaviour change programme.
	Kalichman *et al*., 2009	South Africa (urban) 2 townships 475 men living in two townships in Cape Town	**HIV+: gender-transformative training with men** The five-session intervention emphasized sexual transmission risk reduction and GBV reduction through skills building and personal goal setting, geared towards addressing gender roles, exploring meanings of masculinity and reducing adversarial attitudes towards women. Men were also trained to become vocal advocates for risk reduction behaviour changes with other men in their community. Community randomized trial without control group (Grey scale IIIb)	Men reporting 100% condom use in the past month (or three months – unclear) Men reporting having tested for HIV among men not tested at baseline Men reporting having hit a partner in the past month	Odds ratio at 1 month follow-up: 1.7 (1.1–2.7) Odds ratio at three months follow-up: 0.5 (0.3–0.9) Odds ratio at six months follow-up: 0.3 (0.2–0.4)	Not incremental to basic HIV behaviour change programme.
Community mobilization	**SASA! activist kit for preventing violence against women and HIV** Abramsky *et al*., 2014	Uganda (urban) 4 intervention and 4 control communities Random sample of adult community members sampled at baseline (*n=*1583) and post-intervention (*n=*2532)	**HIV+: gender-transformative community mobilization approach** Community activists were trained, along with staff from selected institutions (e.g. police, health care), to deliver the intervention aimed at changing community attitudes, norms and behaviours related to the power imbalances between men and women that contribute to violence against women and increase HIV risk behaviours. The cadre of activists conducted informal activities within their social networks, using local activism, local media and advocacy, communication materials and/or training. The intervention was not rigidly proscribed but evolved in response to community priorities. Cluster randomized controlled trial (Grey scale II)	Past year concurrent sexual partner among non-polygamous men partnered in the past year	Adjusted risk ratio at four years follow-up: 0.57 (0.36–0.91)	Not incremental to basic HIV behaviour change programme. Due to the movement of trained health and police staff between intervention and control communities, the study examines the added value of the intensive local intervention components, rather than the impact of the whole package.
Mass media	**Mass media GBV and HIV (Soul City)** Goldstein *et al*., 2005	South Africa 2 national surveys of 2000 respondents each, on a sample of African and “coloured” people	**HIV+: integrated GBV messaging** The Soul City series is a multimedia health promotion intervention consisting of a 13-part prime-time television drama, a 45-part radio drama and three basic full colour booklets, distributed through 10 newspapers nationally. Integrated health messages are interwoven and include GBV and HIV/AIDS. Pre-/post-evaluation (Grey scale IIIa)	Proportion of respondents reporting that they always use condoms Control 1 media type 2 media types 3 media types	At eight months follow-up: 6% (*n=*373) 16% (*n=*592) 30% (*n* =522) 38% (*n=*437)	Not incremental to a basic programme, but could be added to a standard HIV mass media campaign. The study analyses the effect of the Soul City series (including HIV and gender messaging) on condom use.
GBV	**Refentse model of comprehensive post-rape services** Kim *et al*., 2009	South Africa (rural) 1 district 207 Survivors of sexual assault (almost exclusively female, average 20 years old – range three months to 94 years)	**Gender+: HIV post-exposure prophylaxis** Provision of voluntary HIV counselling and testing and post-exposure prophylaxis to survivors through a five-part model, including a sexual violence advisory committee, hospital rape management policy, training workshop for service providers, designated examining room and community awareness campaigns Pre-/post-evaluation (Grey scale IIIb)	Completion of 28-day course of PEP drugs	Adjusted risk ratio after 26 months: 3.13 (1.10–8.93)	Not incremental to standard post-rape services. The study analyses the effect of an integrated and comprehensive model of post-rape services on completion of PEP.
	**Stakeholder skills building and awareness raising to prevent GBV among female apprentices** Fawole *et al*., 2005	Nigeria (urban) 350 young female apprentices (203 at follow-up)	**Gender: GBV prevention through stakeholder skills building and awareness raising** The intervention consisted of skills training workshops for apprentices, sensitization training for the instructors of apprentices, police and judicial officers and the development/distribution of educational materials to reduce the incidence of violence. Pre-/post-assessment without control (Grey scale IIIb)	Prevalence of beating Been sexually harassed Seeking judicial redress (or medical care) for rape	At six months follow-up: Dropped from 65.4 to 23% (*p<*0.05) Dropped from 22.9 to 19.7% (*p<*0.05) Increased from 30 to 46% (*p<*0.05)	The study analyses the effect of the intervention on the apprentices’ experience of violence.
Poverty reduction	**Fonkoze Microfinance Programme** Rosenberg *et al*., 2010	Haiti (urban and rural) 34 centres 192 female clients	**Gender: Microfinance loans targeting women** This study assessed the relationship between experience with microfinance loans and HIV risk behaviour among female clients of the Haitian microfinance organization, Fonkoze. Pre-/post-intervention effects (Grey scale IIIa)	Reported that partner was unfaithful Condom use among those with unfaithful partner	Adjusted odds ratios after 12 months: 0.28 (0.13–0.63) 3.95 (0.93–16.85)	The study analyses the effect of accessing microfinance loans on HIV risk behaviour among female clients.
	**Intervention with microfinance for AIDS & gender equity (IMAGE)** Pronyk *et al*., 2006	South Africa (rural) 8 villages Three cohorts of women; 860, 1835 and 3881 women (aged 14 to 35 in last two cohorts)	**Gender+: participatory gender and HIV training curriculum** The sisters for life participatory curriculum of gender and HIV education was facilitated by a team of trainers. The first phase consisted of a structure training (10 sessions done within centre	Experience of IPV in the past 12 months	Adjusted risk ratio after 2 to 3 years: 0.45 (0.23–0.91)	The studies analyse the effect of the combined microfinance and gender/HIV training on IPV, HIV risk behaviours and access to HIV services.
	Pronyk *et al*., 2008	South Africa (rural) 8 villages Sub-group of 262 young women (aged 14 to 35)	meetings every 2 weeks for about six months); and the second phase involved natural leaders being selected, trained and supported to facilitated broader community mobilization. Cluster randomized trial with matched comparison group (Grey scale II)	1) Having accessed voluntary counselling and testing 2) Unprotected sex at last intercourse with a non-spousal partner	Adjusted risk ratios after two years: 1) 1.64 (1.06–2.56) 2) 0.76 (0.60–0.96)	
Social protection	**Zomba cash transfer programme to keep girls in school** Baird *et al*., 2012	Malawi (rural) 2 NGO sites	**Gender: Conditional and unconditional cash transfers for schoolgirls** Cluster randomized controlled trial (Grey scale II)	HIV prevalence and HSV-2 prevalence post-intervention among baseline schoolgirls	Adjusted odds ratios after 18 months: 0.36 (0.14, 0.91) 0.24 (0.09, 0.65)	The study analyses the effect of receiving the cash transfer on prevalent HIV and HSV-2 among girls.
	**Comprehensive school support to adolescent orphan girls** Cho *et al*., 2011	Kenya (rural) 79 households 105 adolescent orphans (Luo students) aged 12 to 14	**Gender:** School support including fees, uniforms, and school supplies. Female teachers were selected and trained as helpers (approximately one helper to 10 participants) to monitor school attendance and intervene as needed, without	Likelihood to delay sexual debut	Logistic regression coefficient at one year follow-up: 1.50	The studies analyse the effect of receiving the school support on early marriage rates and sexual debut.
	Hallfors *et al*., 2011	Zimbabwe (rural) 25 public sites	providing special HIV information or life skills training. Randomized controlled trial (Grey scale II)	Marriage rate	Adjusted odds ratio after two years (*p*≤0.05): 2.92 (1.0, 8.3)	
AIDS education	**School-based provision of information on relative HIV risk** Dupas, 2011	Kenya (two rural districts) 328 primary schools 161 primary schools in TT arm; 71 primary schools in RR arm	**Gender+: informing girls of relative HIV risk** Students in 8th grade were provided with a one-off 40-min information session covering national HIV prevalence by sex and age, and education video about “sugar daddies” and a discussion about cross-generational sex. Cluster randomized control trial (Grey scale II), with difference-in-difference econometric analysis, controlled for school random effects	Incidence of childbearing	At 9 to 12 months follow-up: 1.5% point reduction in childbearing (RR), compared to 5.4% mean in comparison group – i.e. 28% decrease	Not incremental analysis to sexual education in school, which was not effectively being provided at the time of the study, despite an existing national policy. The study analyses the effect of informing girls of relative HIV risk on the incidence of childbearing (proxy of unprotected sex).

a Grey scale I: Strong evidence from at least one systematic review of multiple well-designed, randomized controlled trials; II: Strong evidence from at least one properly designed, randomized controlled trial of appropriate size; IIIa: Evidence from well-designed trials/studies without randomization that include a control group (e.g. quasi-experimental, matched case-control studies, pre-post with control group); IIIb: Evidence from well-designed trials/studies without randomization that do not include a control group (e.g. single group pre-post, cohort, time series/interrupted time series)

b only outcomes on which the intervention had statistically significant effect are included in this table. GBV=gender-based violence; ART=antiretroviral therapy; FSW=female sex worker; IPV=intimate partner violence.

Traditional HIV behaviour change interventions were proven to be enhanced by more gender-responsive approaches, some of which worked with women only, men only or both. Two studies assessed women-focussed HIV risk reduction interventions in South Africa and India that were based on the theory of gender and power, and aimed to build skills in relationship communication and control. Both interventions reduced the rate of reported unprotected sex [65,66], but no effect was found on STI incidence [66]. The former is particularly promising given that the “Raising HIV Awareness in Non-HIV-infected Indian Wives” intervention in India specifically targeted vulnerable women whose husbands engaged in excessive drinking or lifetime physical or sexual spousal violence perpetration [65].

There was evidence of the effectiveness of gender-responsive approaches that promoted a participatory process of critical reflection and debate about gender norms and expectations in relationships. A randomized controlled trial of a participatory gender training with young women and men in South Africa (“stepping stones”) showed a significant incremental impact on HSV-2 incidence, male reported problem-drinking, male soliciting transactional sex and male reported perpetration of violence, although no impact on female reported experience of violence was found [40]. Quasi-experimental evaluations of group interventions with young men, from Brazil, South Africa and India, also found increases in reported condom use and HIV testing, as well as reductions in reported STI symptoms and the reported perpetration of IPV [67–69] .

In addition to approaches targeting enrolled individuals, a community mobilization intervention in Uganda (“SASA!”) seeking to promote community-level change in social norms to prevent violence against women and reduce HIV risk through a community diffusion approach delivered by community activists, led to a significant reduction in sexual concurrency reported by men in non-polygamous relationships and social acceptance of IPV by women [70].

There was also evidence that a multimedia “edutainment” model that integrated the prevention of GBV and HIV messages into a popular soap opera, both increased reported condom use and HIV testing, as well as reduced the acceptance of violence [71,72].

In terms of broader development programmes, a cluster randomized controlled trial (the IMAGE intervention) showed that the addition of participatory gender/HIV training onto an existing microfinance scheme for women in rural South Africa had a significant impact on levels of physical and/or sexual partner violence over two years, and also increased HIV testing and reduced unprotected sex among younger beneficiaries [73,74]. There was also evidence from Haiti suggesting that women's engagement in microfinance alone increased reported condom use and reduced reported numbers of sexual partners [75].

Three effective education-related development programmes were rigorously evaluated in randomized trials. A one-off school-based information session on relative HIV risk profiles by age and sex in Kenya (“sugar daddy talks”) significantly reduced teenage pregnancies [41]. Cash transfers or material support to schoolgirls had significant impacts, including reduced prevalent HIV and HSV-2 [42], reduced marriage rates [76] and delayed sexual debut [77].

Another potentially synergistic intervention for GBV prevention among female apprentices and hawkers in Nigeria suggested the potential to reduce violence in the context of HIV through stakeholder mobilization [78]. Finally, mathematical modelling predicted that adding post-exposure prophylaxis (PEP) for rape victims to GBV programming is an effective adjunct for preventing HIV transmission [79,80].

### Evidence of cost-effectiveness

**Figure 2:**
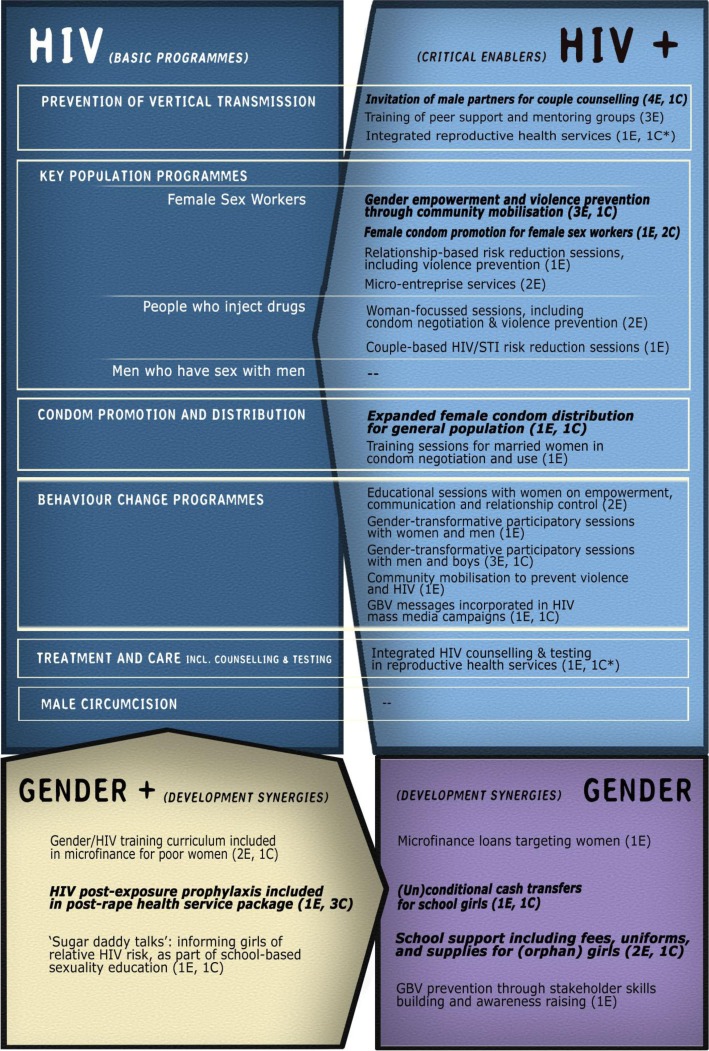
Interventions identified categorized according to the investment framework. Source: Authors (Note: All interventions listed have evidence of effectiveness. The number of effectiveness studies and cost or cost-effectiveness studies are indicated between brackets as E and C, respectively. Bolded interventions have been found to be cost-effective for HIV). *Evidence of the effectiveness and cost-effectiveness of integrated services is from previous systematic reviews.

Our cost and cost-effectiveness search identified 14 publications, including 1 in submission [53] (Table 2). Eleven were cost-effectiveness/cost–utility analyses and three were costing studies [81,82]. Of the 22 effective gender-responsive interventions identified, 11 had a corresponding/comparable costing or economic evaluation (Figure 2). Most only had one costing study, except PEP [80,81,83] and material support to schoolgirls [84,85]. Twelve studies contained data from generalized epidemic settings.

**Table 2:** Summary of costing and cost-effectiveness studies identified

Intervention, study	Setting & target population	Intervention description	Costing scope and methods	Unit cost (2011 US$)	CERs (2011 US$)	Interpretation and limitations
Male involvement through couple counselling for the prevention of vertical transmission John *et al*., 2008	Kenya (urban) 1 ANC clinic 10,000 women enrolled in ANC	HCT included health education, pre-test counselling, testing and post-test counselling. Women attending their first antenatal visit were provided information as a group on HIV-1 infection and vertical transmission prevention interventions, and were then asked to return with their partners after 7 days for HCT. Following pre-test counselling, blood was collected for rapid HIV-1 testing on site and results were disclosed on the same day.	Prospective cohort cost and outcome modelling Incremental financial costing, excluding fixed costs such as rental and utilities Provider perspective Bottom-up costing No sensitivity analysis for cost assumptions	Standard VCT: US$0.84 per woman enrolled in ANC Couple VCT: US$0.90 per woman enrolled in ANC	Standard VCT: US$95.40 per infant infection averted US$16.60 per DALY averted Couple VCT: US$98.45 per infant infection averted US$16.60 per DALY averted	Could be a critical enabler for a vertical transmission prevention programme; or for an HIV testing for treatment programme (HIV+) Highly cost-effective (cost per DALY averted<Kenya's GDP per capita=US$790) Sensitivity analyses found that couple VCT was more cost-effective in scenarios with increased uptake and higher HIV prevalence Outcomes for parents not considered, i.e., HIV infections averted among discordant couples or DALYs averted from ART
Community mobilization and gender empowerment for FSWs Vassall *et al*. (in submission)	India 2 districts 9680 FSWs	This comprehensive HIV prevention programme for high-risk populations had an additional gender-transformative community mobilization component, consisting of the formation of self-help groups, drop-in centres, formation of committees, strengthening of collective action, capacity building, mass events, advocacy and enabling environment.	Empirical, incremental economic costing Modelling of outcomes based on empirical condom use data Provider perspective Combined ingredients approach and top-down Sensitivity analyses conducted for costs	US$18.7– 21 per FSW reached with community mobilization component at least once a year 8.9–19% of the HIV prevention programme was spent on the community mobilization component	US$13.2–19.1 per DALY averted – no ART Cost saving on average with ART	• Could be a critical enabler to a key population (FSW) programme (HIV+) • Highly cost-effective (cost per DALY averted<India's GDP per capita=US$1330) • If ART cost savings are included (assuming 21–40% ART coverage) the intervention becomes cost saving
Female condom programme for commercial sex workers Marseille *et al*., 2001	South Africa Rural population of 3,100,000	A female condom programme serving 1000 commercial sex workers.	Modelled incremental financial costs and HIV treatment cost savings Provider perspective Bottom-up costing Sensitivity analyses for cost assumptions, HIV prevalence among sex workers and clients, and number of clients.	US$0.86 per female condom promoted and distributed (US$0.43–1.72) (US$0.03–0.05 per male condom distributed)^a^ US$5.2 per FSW reached (US$199 per FSW reached)^a^	US$61.28–762.70 per HIV case averted	Could be a critical enabler of a condom promotion and distribution programme; or a FSW programme (HIV+) Highly cost-effective: US$32 (no ART)— 56 (ART) per DALY averted in South Africa<South Africa's GDP per capita=US$6090 (excluding treatment cost savings) Limitations: modelled costs
Female condom promotion and distribution Thomsen *et al*., 2006	Kenya (urban) 1 NGO site 210 FSWs 2382 FSWs (scale-up)	Adding female condom promotion to a male condom programme providing peer education and IEC materials, as well as distributing female condoms.	Empirical (1 & 2) and modelled (3) costs Incremental, financial costing Provider perspective Bottom-up costing	US$305 per participant US$189 per participant (scale-up) US$100 per participant (scale-up, less substitution) (US$29 per FSW reached)^a^	US$4009 per additional consistent condom user US$2559 per additional consistent condom user US$1350 per additional consistent condom user (scale-up, less substitution)	Could be part of condom promotion; key populations; or behaviour change programmes (HIV+) Unclear whether cost-effective, as CER not comparable to international standards, but less cost-effective than male condom promotion and high degree of substitution expected where male condom use is high Limitations: modelled costs
Expanded female condom distribution Dowdy *et al*., 2006	South Africa Brazil Target population not available	Female condom acquisition, distribution, training and education	Modelled costs and outcomes for low, medium and high volumes	US$0.29–1.21 per condom distributed US$0.28–0.82 per condom distributed ((1) US$0.03–0.05 and (2) $0.14–0.25 per male condom distributed)^a^	US$431–1152 per HIV infection averted US$10,287–23,827 per HIV infection averted (Point estimates in different scenarios)	Could be a critical enabler of a condom promotion and distribution programme (HIV+) Highly cost-effective in both countries: US$24 (no ART)– 49 (ART) per DALY averted in South Africa<South Africa's GDP per capita=US$6090; and US$880 (no ART)–1499 (ART) per DALY averted in Brazil<Brazil's GDP per capita=US$9390) Limitations: modelled costs, likely to underestimate demand creation costs
Peer education to transform gender norms Pulerwitz *et al*., 2006	Brazil 2 NGO sites 258 young men 250 young men	Two models: Interactive group education sessions for young men led by adult male facilitators Group education+community-wide “lifestyle” social marketing campaign to promote condom use, using gender-equitable messages.	Empirical, full financial costing Provider perspective Top-down approach	US$158 per participant US$106 per participant US$5.00 per participant per hour of group education ($3.80–6.20) (US$3.40 per employee reached through peer education)^a^	Not available See effectiveness Table (Pulerwitz *et al*., 2006)	• Could be a critical enabler of behaviour change programmes, with gender equity messaging (HIV+) • CERs were not estimated in this study, so it is unclear if it is cost-effective • Limitations: excludes the cost of condoms and other donated inputs, no sensitivity analysis
Mass media edutainment for HIV/AIDS and GBV Muirhead *et al*., n.d.	South Africa Black and coloured adult population (aged 15–49)	The Soul City 4th series was a multimedia edutainment programme producing television drama, radio drama and print materials serialised in 10 national newspapers and booklets around several themes, including HIV/AIDS and violence against women.	Empirical, full economic costing National-level modelling Provider perspective	US$0.04; $0.28 and $0.35 per person reached by radio, print and television US$5.2 million per campaign (40% for Violence against Women theme) (US$12.7 million per HIV mass media campaign)^a^	US$0.56 per weighted effect on HIV-related action ($0.36–0.77)	Could be an enhanced critical enabler with combined HIV and GBV messaging (HIV+) Unclear whether cost-effective, as CER not comparable to international standards 46% (television), 31% (radio) and 34% (print) of total unit cost is for VAW components
HIV post-exposure prophylaxis for survivors of sexual assault Christofides *et al*., 2009	South Africa 2 sites (public facility-based and NGO community-based) Sexual assault survivors	Both models of care provide health and psychosocial support, including a medico-legal examination, HIV testing and counselling, STD treatment, comfort kit, post-exposure prophylaxis therapy for HIV negative survivors. The protocol	Empirical (1) modelling at national level (2, 3) Economic full and incremental costing Provider perspective Mixed bottom-up and	US$819 per survivor ($480–1149) US$402 (full) US$65 (incremental for PEP) (US$29.53 per PEP kit)^a^	US$50,228 net cost per HIV transmission averted without ART ($5924–972,044) US$37,470 net cost per HIV transmission averted with full access to ART ($6833–959,287) US$2311 net cost per life year gained without ART ($272–44,733) US$2149 net cost per life year gained with full access to ART (−$392–55,005)	Could be a development synergy with GBV programmes (Gender+) HIV component=65/402=16% of total, or additional 19% of basic post-rape service package Full intervention is highly cost-effective: US$2120 (no ART)– 2729 (ART) per DALY averted<South Africa's GDP per capita=US$6090
		includes follow-up monitoring visits for counselling, HIV and pregnancy testing and women are supported through the court process.	top-down costing Includes patient-level, site and central-level costs			
Comprehensive post-rape services Kim *et al*., 2009	South Africa (rural) 1 public district hospital 409 rape survivors	Refentse model: five-part intervention model, including the establishment of a sexual violence advisory committee, the formulation of a hospital rape management policy, a training workshop for service providers, designated examining room, and community awareness campaigns.	Empirical, incremental economic costing Provider perspective Mixed top-down (facility-level costs) and bottom-up (patient-level costs)	US$216 per case US$62.60 per case (excl. start-up development costs) (US$29.53 per PEP kit)^a^	Not available	Could be a development synergy with GBV (Gender) Not a cost-effectiveness study Incremental HIV investment is not clearly distinguished from total investment
Comprehensive post-rape services Kilonzo *et al*., 2009	Kenya 3 public health centres 784 rape survivors (43% were children <15 years)	The standard of care included clinical evaluation and documentation, clinical management, counselling and referral mechanisms. Targeted training that was knowledge-, skills- and values-based was provided to clinicians, laboratory personnel and trauma counsellors and coordination mechanisms established with the local police.	Modelled (over one year) Financial costing (excludes start-up capital costs) Provider perspective Top-down	US$30.10 per survivor (US$38.75 per PEP kit)^a^	Not available	Could be a development synergy with GBV (Gender) Not a cost-effectiveness study Incremental HIV investment is not clearly distinguished from total investment Limitations: modelled costs, excludes start-up and capital costs, no sensitivity analysis
Intervention with Microfinance for AIDS & Gender Equity (IMAGE) Jan *et al*., 2011	South Africa (rural) 12 loan centres • 855 poor women in initial two-year trial phase • 2598 poor women in two-year scale-up phase	A gender and HIV training component was added on to a microfinance intervention. The “sisters for life” training curriculum consisted of 10 fortnightly 1-h training and discussion sessions addressing issues such as gender roles, cultural beliefs, relationships, communication, IPV and HIV.	Empirical Incremental, economic costing Provider perspective Ingredients approach	US$50.90 per participant US$15.30 per participant	US$841 per woman with IPV-free year gained US$9107 per IPV-related DALY US$252 per woman with IPV-free year gained US$2733 per IPV-related DALY	Could be a development synergy with economic empowerment interventions for women (Gender+) Unclear whether cost-effective for HIV, as CER is not for an HIV outcome Multiple outcomes not included in CER, i.e., reductions in HIV risk behaviours, increased reported condom use, increased household revenue, improved gender attitudes
Zomba cash transfer programme to keep girls in school Baird *et al*., 2012	Malawi (rural) 2 NGOs 1225 never-married girls aged 13–22 over 18 months	Monthly cash transfers between $4 and 10 provided to households with girls in school or having dropped out at baseline, split between guardian and girl. Conditional group (baseline schoolgirls and dropouts): payment conditional upon 80% school attendance. Unconditional group (baseline schoolgirls): payment received if girl came to the cash point	Empirical Incremental, partial, financial costing Provider perspective Mixed bottom-up (direct) and top-down (administrative costs and fees) costing	US$231 per girl (trial) US$92 per girl (at scale)	US$12,831 per HIV infection averted (trial) US$5132 per HIV infection averted (at scale)	Could be a development synergy with girls’ education or social protection (Gender+) Highly cost-effective at scale cost and with no ART assumption: US$212 per DALY averted (<Malawi's GDP per capita=US$330) Cost-effective in other scenarios (US$365–912 per DALY averted) assuming WHO's upper threshold (<3×GDP per capita=US$990) Multiple outcomes not considered in CERs, i.e., reduced HSV-2 prevalence; reduced teen pregnancies; increased school enrolment and attendance
School support for orphan girls Miller *et al*., 2013	Zimbabwe 183 orphan girls over 3.3 years	School support, including fees, uniforms and school supplies. Female teachers at each intervention primary school were selected and trained as helpers (approximately one helper to 10 participants) to monitor school attendance and intervene as needed, but not to provide special HIV information or life skills training.	Empirical unit costs, modelled ART cost savings and return on education for CER Incremental, economic costing Provider perspective Bottom-up costing	US$1486 per girl (boarders and non-boarders) US$981 girl (non-boarder)	US$6.05 per QALY gained (ranging from −$544 to $2032 per QALY gained in sensitivity analyses)	Could be development synergy with education or social protection (Gender) Highly cost-effective (<Zimbabwe's GDP per capita=US$460), if OVC morbidity is considered an HIV outcome Multiple outcomes considered and monetized on the cost side (return on additional education, ART cost savings)
Education and HIV interventions Duflo *et al*., 2006	Kenya 328 schools in study (240 schools in intervention groups) 70,000 school girls and boys	Three interventions: Training teachers in the HIV/AIDS education curriculum designed for primary schools by the Kenyan government Reducing the cost of education by providing free uniforms Informing teenagers about variation in HIV rates by age and gender	Empirical incremental economic costing Provider perspective Top-down approach	US$5.50 per girl reached US$11.70 per girl reached US$1.70 per girl reached (global range US$11–27 per pupil receiving AIDS education)^a^	US$1006 per pregnancy averted US$863 per pregnancy averted US$105 per pregnancy averted	Could be a development synergy with education, in particular school-based AIDS education and youth programmes (Gender and Gender+) Unclear whether cost-effective for HIV, as CER is not for an HIV outcome Limitations: no detailed cost breakdown, no sensitivity analyses for costs

a Unit costs from the investment framework model (Schwartlander *et al*., 2011) adjusted to 2011 US$. These are only indicated where available in the same unit and where the study identified does not already compare the incremental cost of the intervention. GBV=gender-based violence; GDP=gross domestic product; ART=antiretroviral therapy; CER=cost-effectiveness ratio; FSW=female sex worker; IPV=intimate partner violence.

The studies were of mixed quality, as described in more detail in the Supplementary appendix. Nonetheless, all but two [56,63] contained empirical cost data and half reported economic costs corresponding to the opportunity cost of the investment. Eight studies also provided a cost breakdown of total and/or unit costs. All studies estimated costs from a provider perspective, whereas one also considered societal costs [85]. This could hide considerable patient/participant/community costs, whereby seemingly low-cost interventions may in fact have substantial costs for women and communities. Another important weakness of the cost data is that most estimates are from single sites and small-scale pilots, making it difficult to generalize them at scale.

Seven studies provided CERs in terms of costs per HIV infection averted, HIV DALY averted or HIV QALY gained [42,50,53,56,63,80,84]. This evidence suggests that couple counselling for the prevention of vertical transmission (US$17 per DALY averted); gender empowerment community mobilization for FSWs (US$13–19 per DALY averted); female condom promotion for FSWs (US$32–56 per DALY averted); expanded female condom distribution (US$24–1499 per DALY averted); and PEP for rape survivors (US$2120–2729 per DALY averted) are cost-effective HIV interventions, with CERs well below the respective countries’ GDP per capita (WHO's threshold). By including orphan quality of life as an HIV outcome and various cost scenarios, we find that school support for orphan girls (US$6 per QALY gained) and cash transfers for schoolgirls (US$212–912 per DALY averted) could also be cost-effective in generalized epidemics.

In the absence of relevant CERs in some studies, Table 3 summarizes the estimated additional investment required and the potential additional effect for each gender-responsive intervention with a corresponding basic HIV programme or development programme considered relevant to HIV responses. For example, although couple counselling may cost an additional 7% on standard screening in programmes to prevent vertical transmission, modelling suggests that the changes in uptake combine to avert 3.4% more infant infections [50]. This does not factor in the potential benefits to the parents from knowing their HIV-status and disclosure between couples.

**Table 3:** Incremental costs and effects of gender-responsive interventions for HIV

Programme area	Effective gender-responsive programme components	Cost implications	Additional effect (in bold: effect is from the same study/trial as the cost estimate)	Cost-effectiveness ratio
Prevention of vertical transmission	Facility-based promotion of male involvement in the prevention of vertical transmission through partner notification, partner VCT or couple counselling	7% additional cost per woman in antenatal care [50]	3.4% more infant HIV-1 infections averted [43] 3.4 times more likely to return for follow-up visits and administer nevirapine for delivery [43] 5 times more likely to adopt recommended infant feeding practices [43]	US$16.60 per DALY averted Highly cost-effective
Key populations	Community mobilization to prevent violence against FSWs and promote gender empowerment and leadership	8–24% additional cost per FSW reached [53]	1257–2775 incremental HIV infections averted in two districts in India [53] (but number of infections averted in the basic programme not specified) 1.4 times less likely to report experiencing sexual or physical violence in the past year [51] 34% increase in condom use with clients [52]	US$13.2–19.1 per DALY averted Cost saving (if treatment savings considered) to highly cost-effective
	Female condom promotion and distribution for FSWs	3.4–10.5 times higher unit cost than basic FSW programme [57], compared to the investment framework FSW programme unit cost for Kenya 2.6% additional cost per FSW, compared to the investment framework FSW programme unit cost for South Africa [56]	1.12 times more likely to report consistent condom use with all sexual partners in previous 7 days among FSWs [57] 5.9 HIV infections, as well as 38 syphilis and 33 gonorrhoea cases averted (targeting 1000 FSWs) [56] (but number of infections averted in the basic programme not specified)	US$32–56 per DALY averted (South Africa) Cost saving (if treatment savings considered) to highly cost effective
Condom promotion and distribution	Expanded female condom promotion and distribution	2–3 times higher unit cost than male condoms (Brazil) [63] 10–24 times higher unit cost than male condoms (South Africa) [63]	604 and 9577 incremental HIV infections averted in Brazil and South Africa [63] (but number of infections averted in the basic programme not specified)	US$24–49 per DALY averted (South Africa) US$880–1499 per DALY averted (Brazil) Cost saving (if treatment savings considered) to be highly cost-effective
Behaviour change	Transforming (harmful) gender norms through group education, including men and boys	44 times higher unit cost than behaviour change programmes [68], compared to the investment framework workplace programme unit cost for Brazil	1.19–1.34 times more likely to report condom use at last sex with primary partner^a^ [68] 2 times less likely to report STI symptoms^b^ [68] 33% reduction in HSV-2 incidence [40] (Note: The first two above are combined effects, not the incremental effect of the gender-responsive component, while the latter is the incremental effect above a standard HIV behaviour change programme)	Not available
Mass media	Transforming gender norms for HIV and GBV through multimedia	16% mark-up for the violence against women theme [72], compared to the investment framework mass media campaign cost for South Africa	2.7–6.4^c^ times more likely to report consistent condom use [71] (Note: Combined effect of the HIV/GBV intervention, not incremental effect of the gender-responsive component)	Not available
GBV	Integrated HIV post-exposure prophylaxis in post-rape services	2.2 times higher unit cost than GBV unit cost [80] in the investment framework (for South Africa)	0.6–59.4% reduction in the number of HIV cases estimated as potentially resulting from rape [80] 3.13 times more likely for victims to complete the 28-day course of PEP drugs [82]	US$2120–2729 per DALY averted Highly cost-effective
Education	One-off session for girls on HIV prevalence among older men	6.3–15% additional cost per pupil [41], compared to the investment framework unit cost for AIDS education in schools (global range)	28% decrease in the incidence of childbearing [41]	Not available

a Risk ratios calculated from Pulerwitz *et al*. (2006), based on 148/212 (intervention model 1) and 182/230 (intervention model 2) men reporting condom use at last sex at follow-up, compared to 106/180 men in control group. Risk ratios=1.185 (1.02, 1.378) and 1.344 (1.169, 1.544)

b risk ratios calculated from Pulerwitz *et al*. (2006), based on 53/212 (intervention model 1) men reporting STI symptoms at follow-up, compared to 22/180 men in control group. Risk ratio=2.04 (1.30, 3.23)

c risk ratio calculated from Goldstein *et al*. (2005), based on 271/437 (38%) respondents exposed to three Soul City media types reporting always using condoms, compared to 22/373 (6%) not exposed to any Soul City media; and 95/592 (16%) respondents exposed to one Soul City media type reporting always using condoms, compared to the same control. GBV=Gender-based violence; FSW=female sex worker.

Overall, these examples suggest that investment in strengthening the gendered components of projects – though adding costs over and above basic programme costs – could generate significant additional HIV benefits. Some may even be cost-saving, especially if averted treatment costs are considered. Such interventions include gender-responsive community mobilization for FSWs, female condom promotion for FSWs and PEP for rape survivors. It also appears that although female condom promotion (including for FSWs) and PEP may be highly cost-effective, they may be considerably under-budgeted in global resource needs estimates.

Unfortunately there was insufficient data to determine whether the remaining gender-responsive interventions, though effective, are good value for money. For example, although the unit costs of interventions working with young men to transform gender norms seem reasonable (US$106–158 per participant in Brazil), it was not possible to assess their HIV-related incremental effects or cost-effectiveness.

## Discussion

This is the first study to systematically review the costs and cost-effectiveness of gender-responsive interventions for HIV. We identified a range of interventions with demonstrated impacts either on HIV or its proximate determinants. Most appear to function as the investment framework's critical enablers of basic HIV programmes, although several examples of development synergy type interventions were also identified (Figure 2). The strategies identified include transformative interventions that influence the dynamics and balance of power within relationships; collectivization and peer support empowerment strategies; women-focussed interventions providing education, skills training and the development of self-efficacy; integrated gender and HIV behaviour change through multimedia, community mobilization or trainings that include men and boys; and economic support to poor women, FSWs and schoolgirls. However, many of these interventions had no economic analyses, making it difficult to assess their cost-effectiveness.

Where cost data were available, among the interventions found to be cost-effective, enrolling couples in programmes to prevent vertical transmission seems to help reduce the inefficiencies associated with high loss to follow-up along the service cascade [86,87]. Because counselling and testing is an entry point into ART, the only incremental cost would be that of generating male demand for counselling and testing in antenatal clinics [88]. The involvement of male partners is likely to be more cost-effective in high prevalence settings with higher uptake of couple counselling [50]. However, there may also be real risks of aggressively promoting such an approach, if it becomes a barrier to service access for single women, for example, or puts women in violent relationships at further risk [89]. For this reason, it will be important to assess whether such initiatives have unforeseen negative consequences that undermine their utility, and consider whether additional programmatic elements, to potentially mitigate this risk, may be needed.

Similarly, to be a more attractive investment, female condom promotion may require intensified demand creation through woman-focussed, transformative programmes and/or subsidised distribution. Although it is already considered a basic HIV programme and modelled studies found that it was cost-effective [56,63], it is currently not included in the investment framework for most countries with generalized epidemics [12] – possibly because of low demand coupled with high commodity costs. The cost of additional demand creation activities and their effectiveness at creating new demand among vulnerable women (rather than substituting demand for male condoms) will greatly influence the intervention's value-for-money [56,57]. Not surprisingly, concentrating on women at greatest HIV risk appears to be particularly cost-effective [56].

Evidence from India suggests that gender-responsive community mobilization activities aiming to promote collectivization are highly cost-effective critical enablers to basic FSW programmes [53]. Additional research is needed to assess whether this model can be adapted and implemented cost-effectively in generalized epidemic settings.

Evidence from South Africa suggests that PEP for rape survivors is cost-effective [80] and an indisputable intervention to prioritize from a human rights perspective. Its cost-effectiveness will depend on the underlying HIV prevalence among survivors and perpetrators, its early administration and adherence/completion rates [82].

The evidence from the IMAGE study of the impact of combined economic empowerment and gender/HIV training illustrates the potential to add HIV-specific components onto livelihood programmes for women, leveraging the development synergy, at low incremental cost.

Certain GBV prevention interventions could function as both critical enablers as well as development synergies. For example, gender-transformative activities focussing on young men could either be classified as critical enablers, where the gender norms component is integrated in standard group HIV behaviour change programmes; or as development synergies, where an HIV component is added to a broader gender transformation programme. This would have implications for their cost-effectiveness, as it would determine what such additional investments are incremental to. Similarly, mass media campaigns are already considered critical enablers of HIV behaviour change; hence, the more explicit inclusion of GBV prevention messaging could be considered a gender-responsive critical enabler. However, where such a multimedia campaign exists with a focus on GBV and gender transformation, this again represents an opportunity to leverage related development resources.

There is evidence that other gender-responsive development programmes can also influence HIV-related outcomes through structural pathways and still be cost-effective for HIV. The provision of cash transfers to keep girls in school [42,90], for example, could be cost-effective for HIV under certain conditions. Although such interventions could also be considered development synergies, they currently are not costed in the investment framework. This suggests that the scope of development synergies should be broadened, to consider potential investment options that are likely to achieve both HIV and non-HIV outcomes, and merit co-financing, instead of focussing primarily on HIV-specific activities [91].

Although we did not review evidence on the impact and costs of the integration of sexual and reproductive health and HIV services, this area does merit consideration as a gender-responsive enabling approach, as it restructures services to better meet women's needs. A systematic review reported consistent and multiple HIV-related benefits from a range of integration models, including reduced HIV incidence [92]. Another review found that integrated programmatic approaches could lead to efficiency gains through economies of scale and scope, and be cost-effective or cost-saving, especially where HIV counselling and testing was integrated into family planning or family planning was integrated in services to prevent vertical transmission [33].

It is worth noting that the review did not identify any (cost-) effectiveness studies of gender-responsive components linked to ART, male circumcision or programmes for men who have sex with men and their female partners. Yet, gender inequalities in these areas underscore the need to ensure their gender-responsive delivery, particularly given the important share of HIV budgets that they claim with about 50% of HIV spending going to treatment and care across low- and middle-income countries [93,94].

This review has several limitations. Fundamentally, there is limited evidence on the costs and cost-effectiveness of gender-responsive interventions; and even fewer analyses of how gender-specific components may serve to increase the impact of basic programmes, or the potential impacts of HIV-relevant components in gender-specific development programmes. These are important areas for further investigation. We have included effectiveness studies even where cost/cost-effectiveness data was lacking in an attempt to highlight these gaps and recommend them as key opportunities for additional economic research.

We decided to include self-reported behavioural outcomes when considering intervention effectiveness, despite the known challenges of these indicators as proxies of behaviour change and programme effectiveness. The lack of availability of intervention evaluations with biological outcomes is not unique to this field, and the resulting conclusions need to be interpreted with caution. However, we felt that it was preferable to include such evidence as a starting point for programming, rather than have a potentially overly narrow focus of intervention evidence.

Although we were interested in incremental investments over and above basic programmes, not all retained studies actually measured this incremental cost/effect. For example, the GBV and HIV messaging in the Soul City multimedia campaign were fully integrated and it was not possible to isolate the GBV component's effect [72]. In such cases, we have been cautious with our conclusions and reflected this uncertainty.

Another important limitation is the extent to which single or a small number of studies per intervention have external validity. Moreover, the quality of the cost and effectiveness data was mixed, with particularly little consistency in outcomes measured and costing methods. This hampered any interpretation of the cost-effectiveness of gender-responsive training sessions with young men, for example.

Publication bias could also imply that we are overestimating effectiveness and by only considering interventions with quantifiable evidence of effect, we may have excluded important qualitative evidence, as well as complex interventions that are not amenable to experimental designs 95–98] . Other promising interventions were excluded, because they did not include an explicit HIV focus [99–101] .

## Conclusions

Despite the critical role of gender inequality and GBV in fuelling the HIV epidemic and impeding service effectiveness [7,11], there is a paucity of evidence on the (cost-) effectiveness of specific programme components to address women's needs and transform harmful gender norms for HIV impact. Where available, evidence suggests that gender-responsive interventions, including GBV prevention, can function as cost-effective critical enablers by addressing harmful gender norms and thus improving HIV service uptake, adherence and behaviour change. Indeed, our review identified a number of promising cost-effective gender-responsive interventions that merit consideration as critical enablers in the investment framework, as well as others that may be better placed as development synergies.

Given the many data gaps, these interventions are by no means an exhaustive package, but rather the first set of promising interventions in a neglected field. Countries may want to consider these interventions for gender-responsive HIV programming. Indeed, the absence of cost-effectiveness data should not be interpreted as evidence that these investments are not good value-for-HIV-money. More research is needed to establish the return on these investments, in terms of increased utilization of and adherence to HIV services, and ultimately infections and deaths averted, especially for interventions known to be effective.

The findings of this study have important financing implications. Although cost data are limited and the type of interventions too heterogeneous to determine a standard cost benchmark, the compiled evidence appears to justify an additional allocation of at least 5%, over and above the current 10% markup for critical enablers, to accommodate activities that address gender equality and GBV. This could initially require an overall increase in resource needs for the global HIV response, but it is likely that this investment could reduce future treatment costs and increase programme efficiency. Reallocating current spending may also be considered. That being said, there is increasing commitment from donors to make high-impact gender-responsive investments. For example, the Global Fund's new funding model requirements include a strong focus on gender-responsive programming and also encourage the use of grants to fill gaps relating to the cost of priority interventions that address gender inequality [102].

Interventions that tackle the structural drivers of HIV, such as inequalities in access to education, poor livelihoods and harmful gender norms commonly have multiple societal benefits, including for HIV. As such, the HIV sector should co-finance these interventions with other sectors, to ensure that all benefits are generated [91,103] and other development resources fully leveraged [104]. Given existing resource constraints, there will be growing emphasis on ensuring that available resources are used to best effect. Including cost-effective, gender-responsive interventions in HIV portfolios will likely increase programme efficiency, as well as advance gender equity for more sustainable responses.

## Supplementary Material

The cost and cost-effectiveness of gender-responsive interventions for HIV: a systematic review
