# Supplementary material for: The cost and cost-effectiveness of gender-responsive interventions for HIV: a systematic review
Source: J Int AIDS Soc. 2014 Nov 4;17(1):19228. doi: 10.7448/IAS.17.1.19228 (PMC4221500; doi:10.7448/IAS.17.1.19228)
Supplement: The cost and cost-effectiveness of gender-responsive interventions for HIV: a systematic review [file JIAS-17-19228-s001.pdf]

The cost and cost-effectiveness of gender-focussed interventions for HIV: a systematic review

Remme M, Siapka M, Vassall A, Heise L, Jacobi J, Ahumada C, Gay J & Watts C

SUPPLEMENTARY APPENDIX

Conceptual framework

We used an established classification to help categorise interventions along a 'gender continuum', according to the level of change that they seek to achieve, ranging from being gender-negative, gender-blind or gender-neutral; through to being gender-sensitive; or gender-transformative(1-2).

A gender-negative, blind or neutral intervention may aggravate, reinforce or ignore existing gender inequalities and norms, as it would not acknowledge the different needs of women and men.

A gender-sensitive intervention takes into account these differences in its design and attempts to ensure that women and/or men will benefit from the intervention. A relevant example is the inclusion of the female condom in condom promotion activities, to try to address the challenges that women may have in negotiating male condom use.

A gender-transformative intervention would explicitly seek to redefine and transform gender norms and relationships in order to redress existing inequalities. For example, an intervention that sought to challenge assumptions and communication about gender roles that make it difficult for women to negotiate condom use in the first place, and encourage them to take the initiative and protect themselves by using female condoms, would be gender-transformative. In both of these cases, the programmes would have a gender-focussed intervention component, but with different ambitions regarding the extent of change that it is seeking to achieve.

For this review, we further grouped the interventions identified into four types (Figure X1). The first are basic HIV programmes from the *Investment Framework* (3) delivered in a gender-sensitive manner. The second are gender-sensitive or gender-transformative activities that can be added to basic programmes to enhance their effectiveness and efficiency by addressing gender-related barriers to behaviour change, service uptake and retention (HIV+ or critical enablers). The third comprises HIV-specific interventions that could be added onto gender-transformative development programmes, to achieve a synergistic HIV effect (Gender+). The last type (Gender) consists of gender-transformative development interventions that do not explicitly include programmatic HIV components, but may nevertheless have secondary HIV benefits due to their impact on gender inequalities.

Figure X1. Conceptual framework - Types of gender-responsive interventions and programme components that could be considered within HIV investment approaches

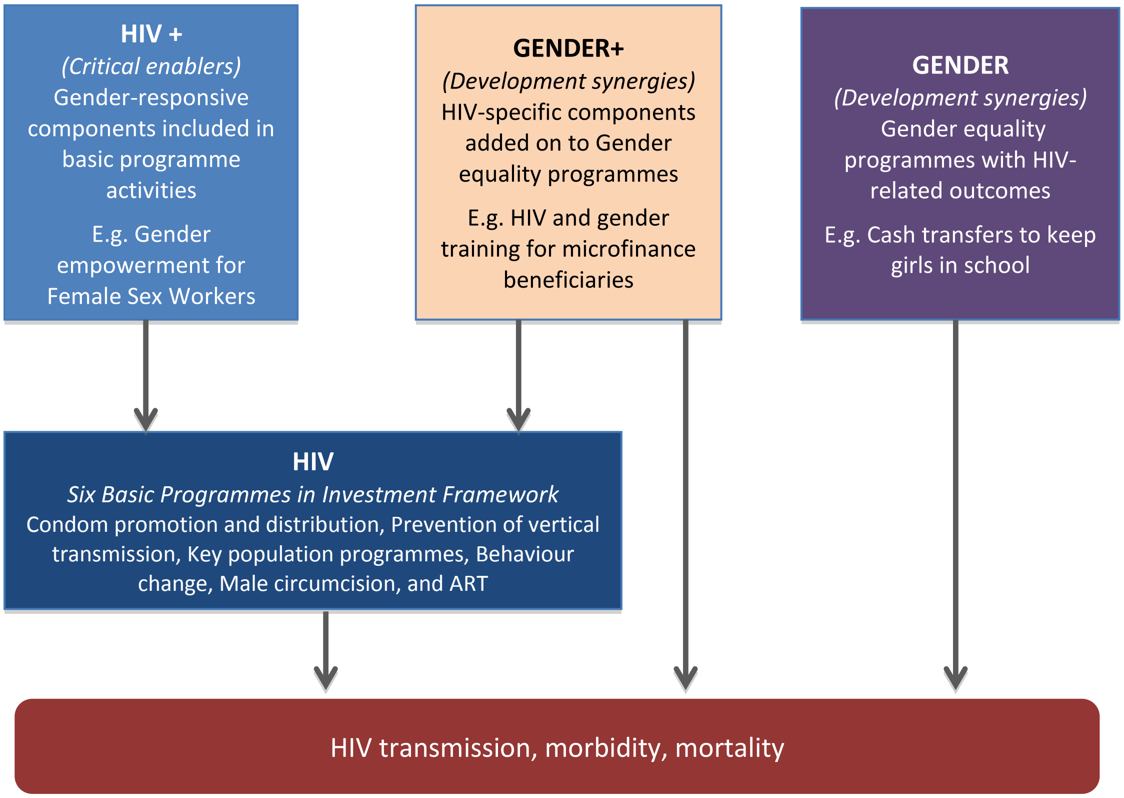

***Source**: Authors*

Pathways

The ultimate HIV outcomes we considered are the ones in the UNAIDS *Investment Framework*, i.e. HIV infections averted (transmission), AIDS-related deaths averted (mortality) and disability or quality-adjusted life years saved (morbidity). In practice, however, few studies were designed to assess impact on HIV or other biological outcomes. The majority focussed on proximal determinants of HIV risk, such as condom use or other reported behaviours. These intermediate outcomes lie along the causal pathway to HIV impacts, and so if a significant change has occurred, it is likely that this would ultimately lead to a reduction in HIV vulnerability.

Figure X2 illustrates the hypothetical causal pathways that we formulated connecting these intermediate outcomes to HIV impacts.

To ensure that we were in line with the *Investment Framework*, we did not consider impact mitigation as an end objective of an HIV response. Hence, interventions to mitigate the impact of HIV on affected households and communities were excluded, although a large burden of care for people living with HIV and orphans is disproportionately borne by women and girls(4).

Figure X2. Potential pathways between gender inequality, violence and HIV vulnerability

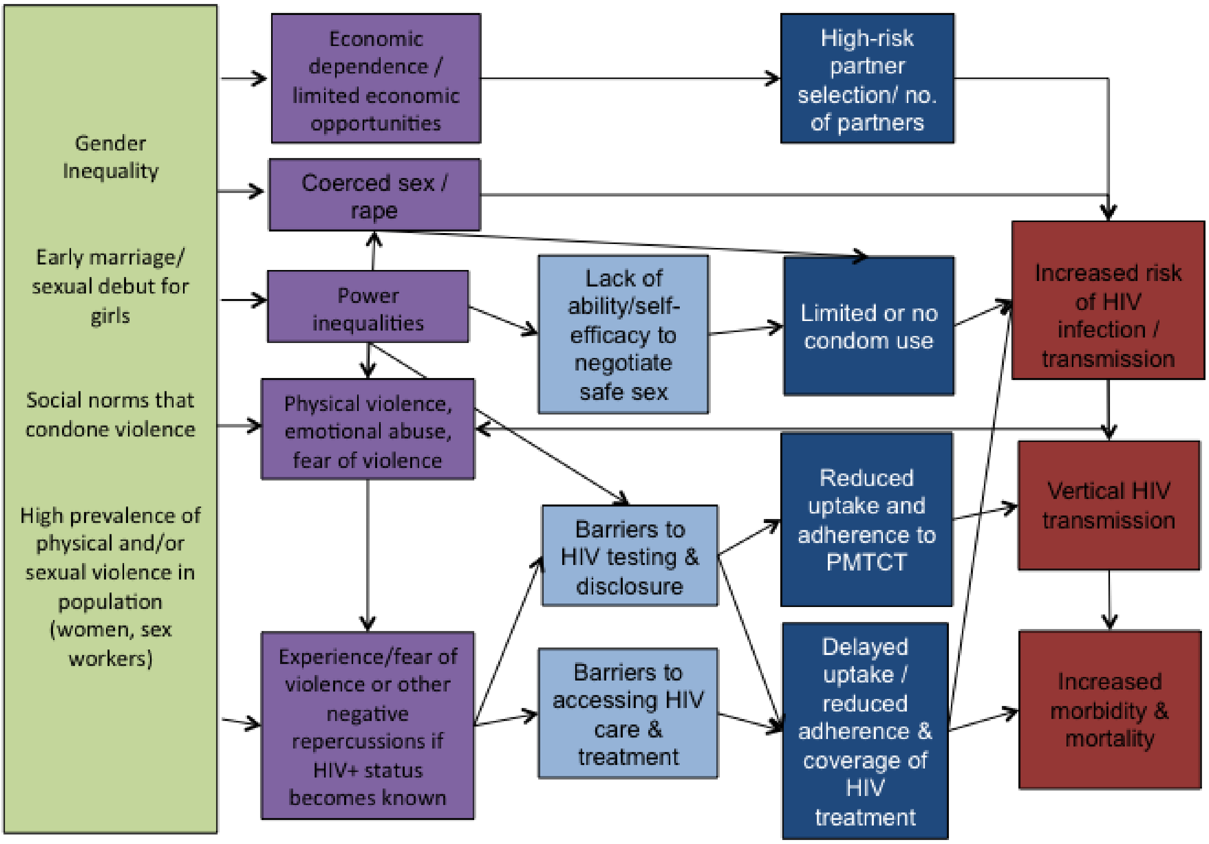

Source: Authors

Search Strategy

To identify all published costing and economic evaluation studies of gender-focussed HIV interventions, we searched PubMed, EconLit, Eldis and HIV and gender websites, following PRISMA guidance(5). The terms searched thematically covered (1) HIV/AIDS; (2) gender; (3) intervention; and (4) economic/impact evaluation.

The full PubMed search string was: AIDS[Title/Abstact] OR HIV OR Acquired Immunodeficiency Syndrome AND Cost OR Cost effectiveness[Title/Abstract] OR cost analysis[MeSH Terms] OR cost benefit[MeSH Terms] OR cost benefit analysis OR cost effectiveness analysis OR effect*[Title/Abstact] OR impact AND livelihood OR skills OR training OR literacy OR violence AND Gender[Title/Abstract] OR women[MeSH Terms] OR female OR masculinity OR empowerment AND Intervention[Title/Abstract] OR program* OR project OR service OR community mobilisation OR mass media OR microfinance OR family planning OR sexual and reproductive health.

Figure X2 summarises the results from the effectiveness and cost/cost-effectiveness search, including both peer-reviewed and grey literature.

Quality assessment and Results

The quality of the costing and economic evaluation studies was assessed using an adapted version of the British Medical Journal's checklist for economic evaluations(6). In addition to the standard 35 items, we included seven that we considered relevant, i.e. economic costing; empirical costs; costs from more than one site; breakdown of costs provided; all relevant cost categories included; sensitivity analyses conducted for costing assumptions; and cost-effectiveness ratios presented by QALY/DALY. Each study that reported unit cost data only was considered a costing study and assessed against a potential 29 items, whereas studies reporting a cost-consequence ratio were assessed as economic evaluations (42 items). Two reviewers scored study quality independently and discrepancies were resolved through discussion.

Figure X3 summarises study quality based on the number of studies with a certain range of limitations identified. While none of the studies were flawless or reported all of the information required, five economic evaluations were of very good quality, with 5-9 limitations (out of 42). The remaining economic evaluations were spread evenly between 10-14 limitations (2 studies); 15-19 limitations (2); more than 20 limitations (2). Two of the education-related economic evaluations were in the latter category, mostly because they provided very limited information on their costing methods. The three costing studies were of similar quality (12-14 limitations out of 29), with limited reporting of methods applied, financial costing (rather than economic costing) and no sensitivity analyses of cost assumptions, among others.

Figure X3. Study Quality: Number of Limitations per Study using a modified version of the BMJ quality assessment checklist for economic evaluations

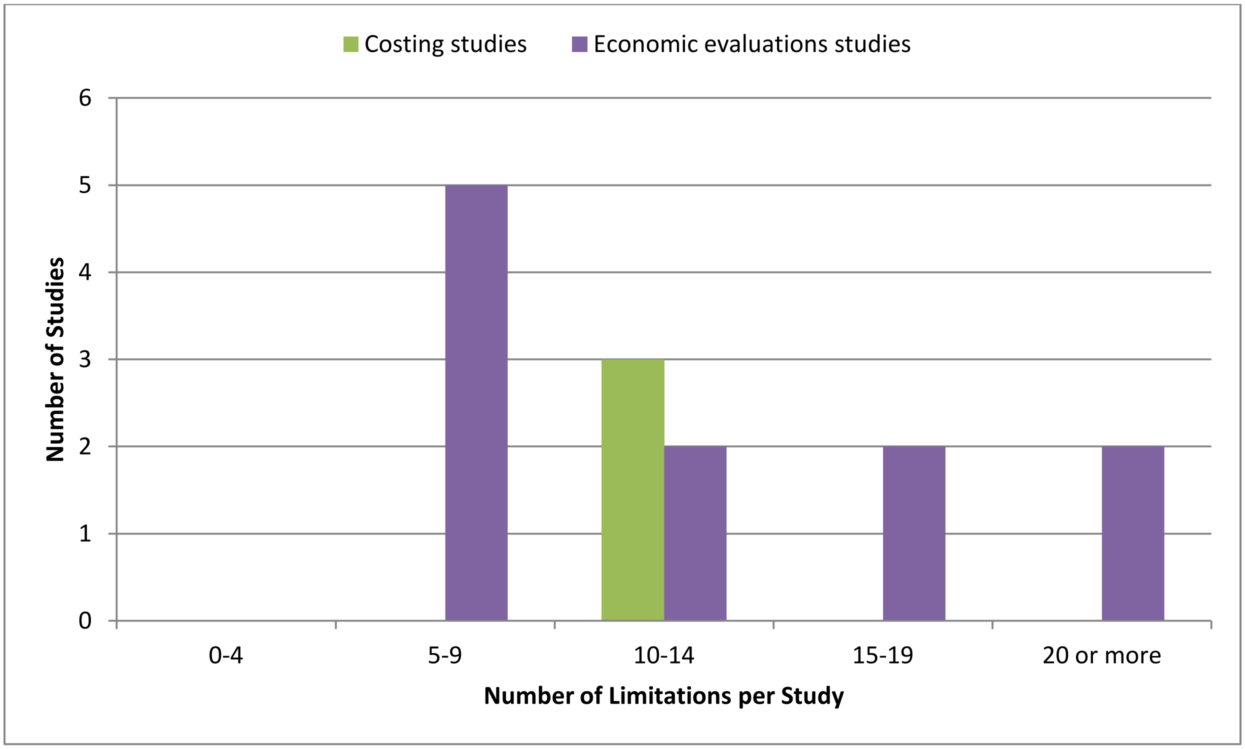

Figure X4. Incremental costs and effects of Gender-focussed interventions for HIV on a Cost-effectiveness plane

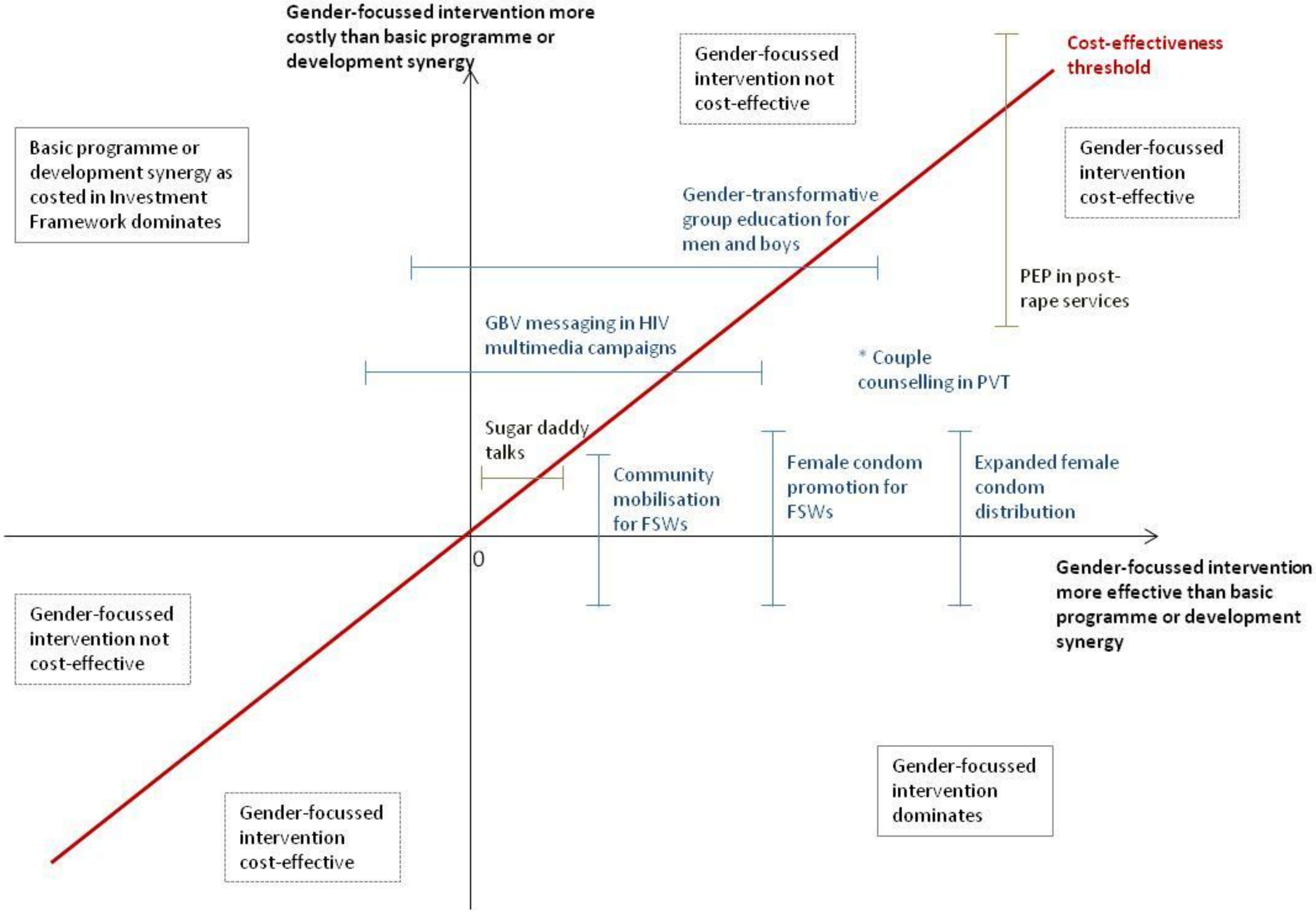

Source: *Authors*

References

1. Kambou S, Magar V, Gay J, Lary H. Walking the Talk: ISOFI, A Gender and Sexuality Initiative. Washington, DC: International Center for Research on Women2006.

2. UN INSTRAW. Glossary of Gender-related Terms and Concepts. United Nations International Research and Training Institute for the Advancement of Women; n.d. [28 October 2012]; Available from: http://unamid.unmissions.org/Default.aspx?tabid=11012&language=en-US.

3. Schwartlander B, Stover J, Hallett T, Atun R, Avila C, Gouws E, et al. Towards an improved investment approach for an effective response to HIV/AIDS. Lancet. 2011 Jun 11;377(9782):2031-41.

4. UNAIDS. Agenda for Accelerated Country Action for Women, Girls, Gender Equality and HIV. Operational plan for the UNAIDS action framework: addressing women, girls, gender equality and HIV.n.d.

5. Moher D, Liberati A, Tetzlaff J, Altman DG. Preferred reporting items for systematic reviews and meta-analyses: the PRISMA statement. PLoS Med. 2009 Jul 21;6(7):e1000097.

6. Drummond MF, Jefferson TO. Guidelines for authors and peer reviewers of economic submissions to the BMJ. The BMJ Economic Evaluation Working Party. BMJ. 1996 Aug 3;313(7052):275-83.
